# Supplementary material for: Hexokinase-I directly binds to a charged membrane-buried glutamate of mitochondrial VDAC1 and VDAC2
Source: Commun Biol. 2025 Feb 10;8:212. doi: 10.1038/s42003-025-07551-9 (PMC11811193; doi:10.1038/s42003-025-07551-9)

Supplementary Information

Hexokinase-I directly binds to a charged membrane-buried glutamate of mitochondrial VDAC1 and VDAC2

Sebastian Bieker[1,2]*, Michael Timme[1,2]*, Nils Woge[1,2], Dina G. Hassan[1,2,3], Chelsea M. Brown[4], Siewert J. Marrink[4], Manuel N. Melo[5#], Joost C. M. Holthuis[1,2#]

[1]Molecular Cell Biology Division, Department of Biology/Chemistry, University of Osnabrück, 49076 Osnabrück, Germany

[2]Center for Cellular Nanoanalytics, Osnabrück University, Artilleriestraße 77, 49076 Osnabrück, Germany

[3]Department of Environmental Medical Sciences, Faculty of Graduate Studies and Environmental Research, Ain Shams University, Cairo, Egypt

[4]Groningen Biomolecular Sciences and Biotechnology Institute, University of Groningen, Nijenborgh 7, 9747 AG Groningen, The Netherlands

[5]Instituto de Tecnologia Química e Biológica António Xavier, Universidade Nova de Lisboa, Av. da República, 2780-157 Oeiras, Portugal

*These authors contributed equally

[#]Corresponding authors: m.n.melo@itqb.unl.pt and holthuis@uos.de

This PDF file includes:

Supplementary Figs. 1 to 8

Supplementary Movies 1 and 2

Supplementary Tables 1 to 3

Image J Macro

Unprocessed images of immunoblots

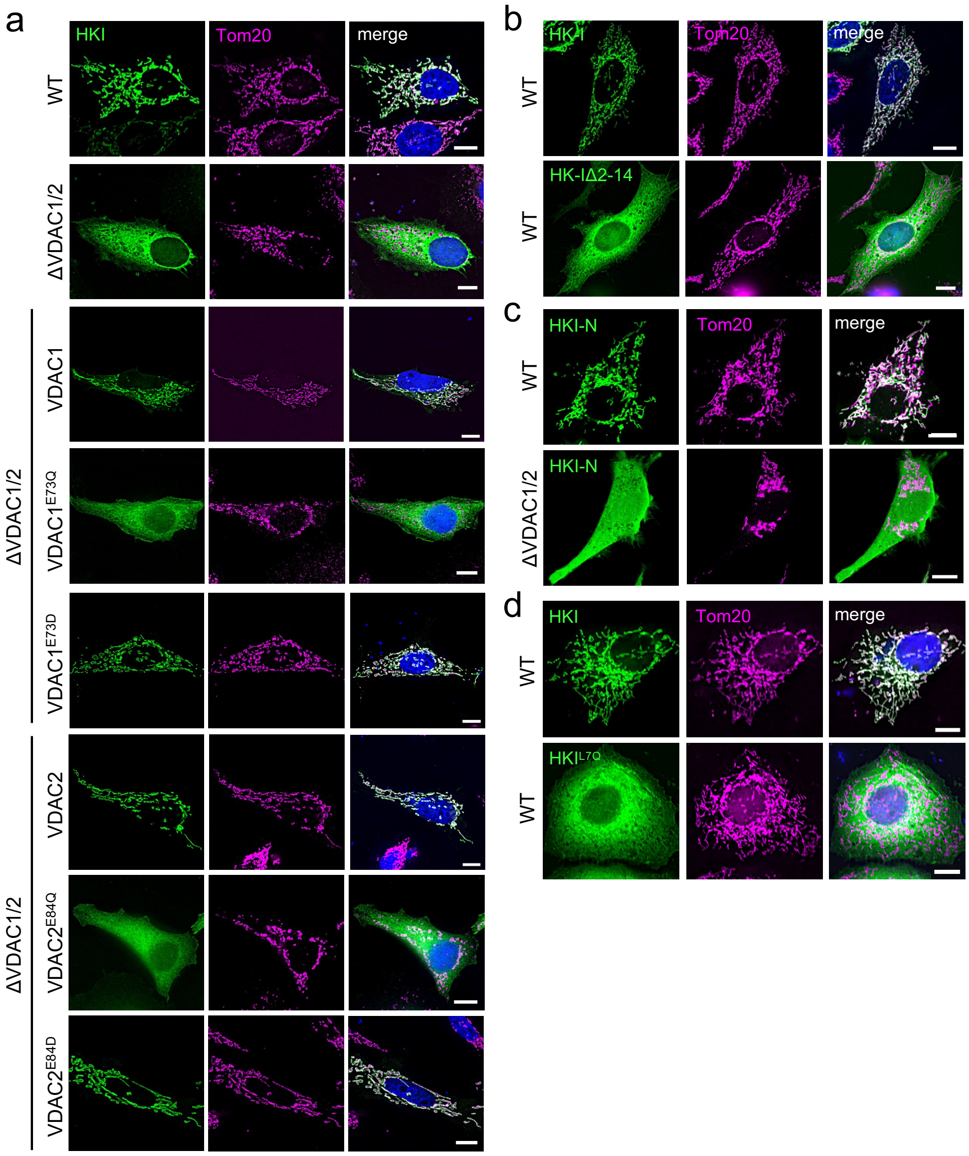

Supplementary Figure 1 | Mitochondrial localization of HKI relies on its N-terminal α-helix and a membrane-buried Glu in VDACs. (**a**) Fluorescence images of wild-type (WT) and VDAC1/2-DKO HeLa cells expressing EGFP-tagged HKI (*green*) alone or in combination with HA-tagged VDAC1, VDAC1^{E73Q}, VDAC1^{E73D}, VDAC2, VDAC2^{E84Q} or VDAC2^{E84D}, fixed and then stained with DAPI (*blue*) and an antibody against Tom20 (*magenta*). Scale bar, 10 μm. (**b**) Fluorescence images of WT HeLa cells expressing EGFP-tagged HKI or *N*-terminal truncation mutant HKI$_{\Delta 2\text{-}14}$, fixed and then stained with DAPI (*blue*) and an antibody against Tom20 (*magenta*). Scale bar, 10 μm. (**c**) Fluorescence images of live WT and VDAC1/2-DKO HeLa cells co-expressing EGFP-tagged Tom20 (*magenta*) and Halo-tagged HKI-N (*N*-terminal HKI residues 1-17, *green*). Scale bar, 10 μm. (**d**) Fluorescence images of WT HeLa cells expressing EGFP-tagged HKI or HKI^{L7Q} (*green*), fixed and then stained with DAPI (*blue*) and an antibody against Tom20 (*magenta*). Scale bar, 10 μm.

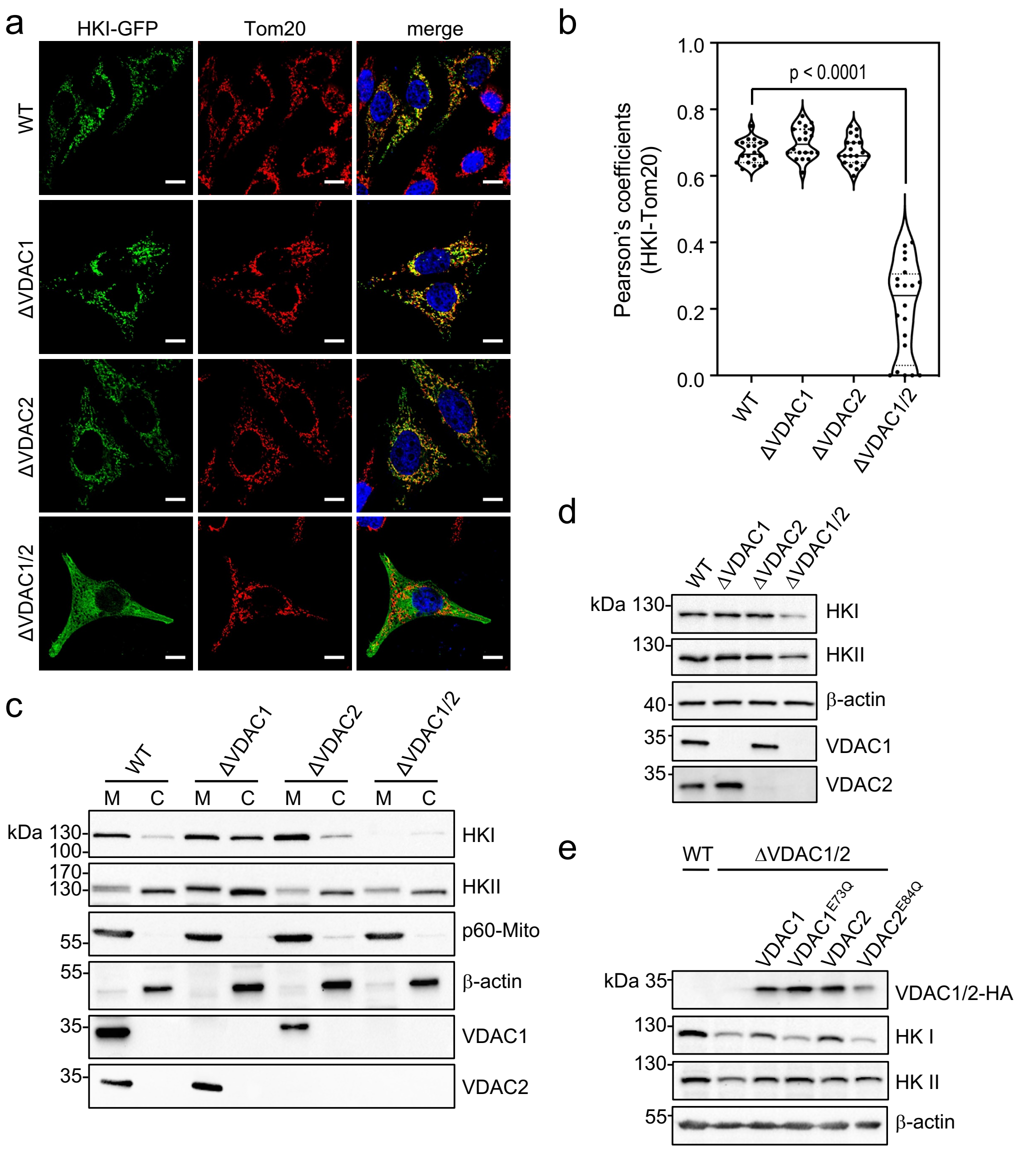

Supplementary Figure 2 | Both VDAC1 and VDAC2 contribute to stabilizing the mitochondrial pool of HKI. (**a**) Fluorescence images of wild-type (WT) and VDAC1-KO, VDAC2-KO and VDAC1/2-DKO HeLa cells expressing EGFP-tagged HKI (*green*) fixed and then stained with DAPI (*blue*) and an antibody against Tom20 (*red*). Scale bar, 10 μm. (**b**) Pearson's correlation co-efficient analysis between HKI and Tom20 signals in cells as in (a). Data shown are based on the analysis of 20 cells per condition from two independent experiments. *p* values were calculated by unpaired two-tailed *t* test. (**c**) WT, VDAC1-KO, VDAC2-KO and VDAC1/2-DKO HeLa cells were subjected to subcellular fractionation. Mitochondrial (M) and cytosolic fractions (C) were analysed by immunoblotting with antibodies against VDAC1, VDAC2, HKI, HKII, p60-Mito and β-actin. (**d**) Total cell lysates from WT, VDAC1-KO, VDAC2-KO and VDAC1/2-DKO human HCT116 cells were subjected to immunoblotting with antibodies against VDAC1, VDAC2, HKI, HKII and β-actin. (**e**) Total cell lysates from WT and VDAC1/2-DKO human HCT116 cells transduced with HA-tagged VDAC1, VDAC1^{E73Q}, VDAC2 or VDAC2^{E84Q} were subjected to immunoblotting with antibodies against the HA-tag, HKI, HKII and β-actin. Immunoblots stained with anti-β-actin, anti-VDAC1 and anti-VDAC2 antibodies in (d) and (e) are identical to those shown in Suppl. Fig. S10 of Dadsena et al.[1]

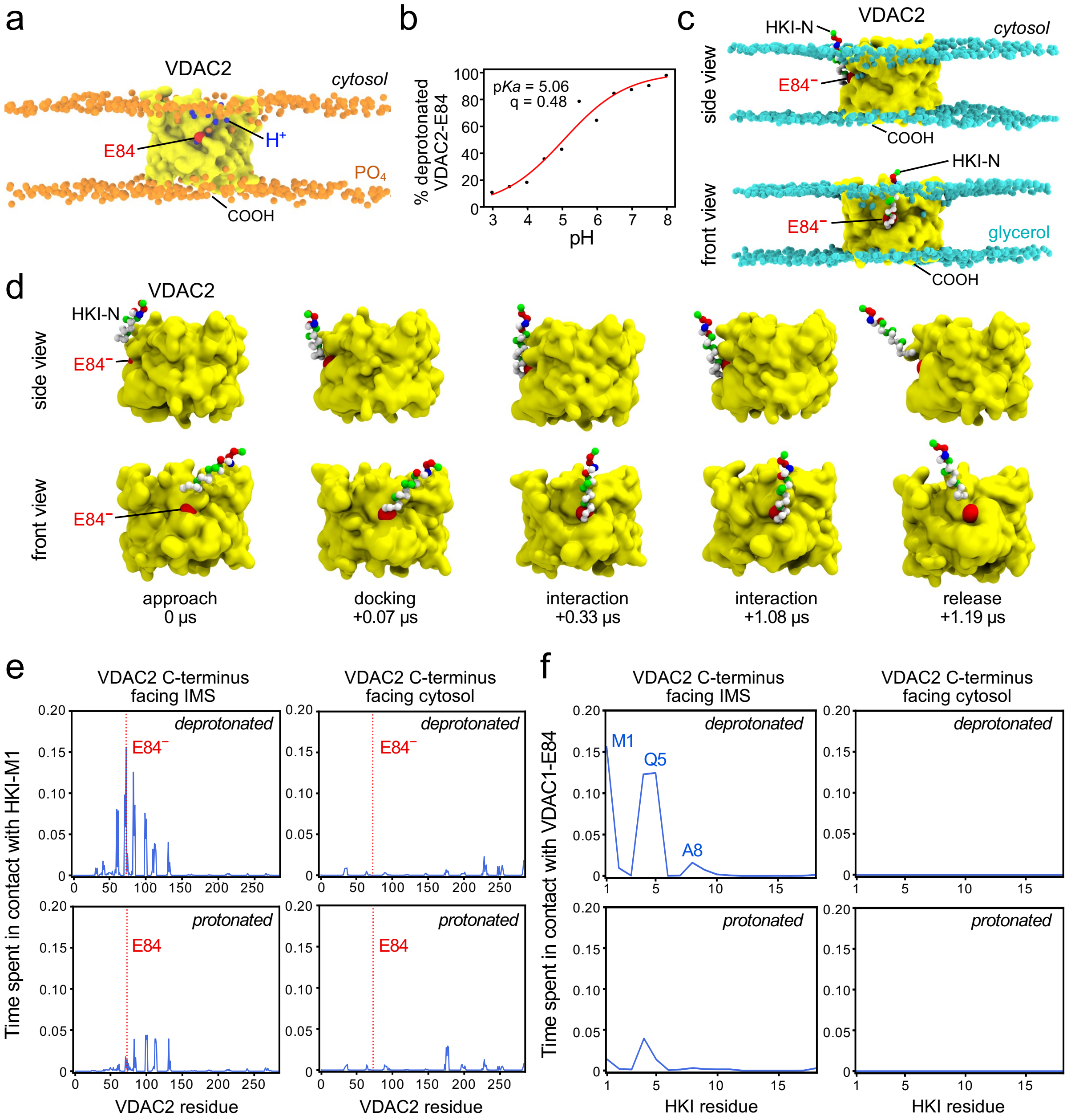

Supplementary Figure 3 | HKI-N binding to VDAC2 is directly controlled by the protonation state of the membrane-buried Glu. (**a**) Still from a titratable MD simulation of VDAC2 (*yellow*) to evaluate the protonation state of E84 (*red*) at pH 5.0. PO_4 groups in the POPC-based bilayer are marked in *orange* and protons are marked in *blue*. (**b**) Titration curve showing the degree of deprotonation of E84 in VDAC2, simulated at a pH range of 3-8. (**c**) Stills from an MD simulation showing HKI-N bound to VDAC2 with a deprotonated E84 (*red*) and IMS-facing *C*-terminus. Glycerol groups in the OMM-mimicking bilayer are marked in *cyan*. (**d**) Stills from an MD simulation, showing the approach and binding of HKI-N to VDAC2 with a deprotonated E84 (*red*) and IMS-facing *C*-terminus. (**e**) Relative duration of contacts between HKI-Met1 and specific residues of VDAC2 with a protonated or deprotonated E84 and cytosol- or IMS-facing *C*-terminus. Shown are the combined data of three individual replicas with a total simulation time between 168 µs and 177 µs per condition. (**f**) Relative duration of contacts between VDAC2-E84 and specific residues of HKI-N under the same conditions as in (e).

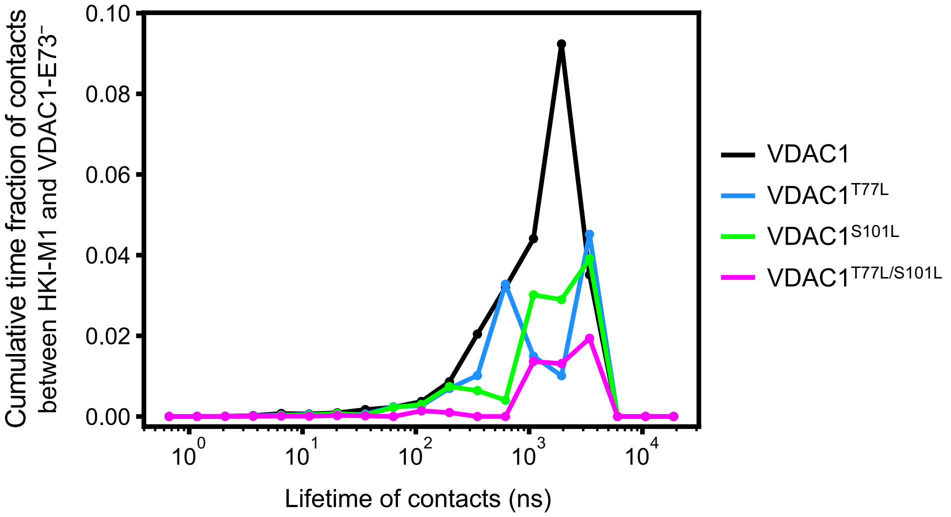

Supplementary Figure 4 | Distribution of the HKI-Met1 contact lifetimes with deprotonated Glu73 of VDAC1, VDAC1^{T77L}, VDAC1^{S101L} and VDAC1$^{T77L/S101L}$. Binding events over all replicates were binned according to their lifetimes. The resulting histograms were then weighted by the summed lifetimes of the events in each bin. Finally, values are presented normalized by the total simulation time of each system's replicates. The different mutants have lower overall binding affinity than wildtype VDAC1, as evidenced by lower histogram values, but retain similar average binding times (weighted average bound lifetimes of 1.50±0.18 μs, 1.88±0.49 μs, 1.80±0.39 μs and 2.22±0.61 μs for VDAC1, VDAC1^{T77L}, VDAC1^{S101L} and VDAC1$^{T77L/S101L}$, respectively). This indicates that the reduced binding is a result of a lower on-rate, rather than shorter contact lifetimes — the converse would have entailed a distribution shift to lower lifetimes.

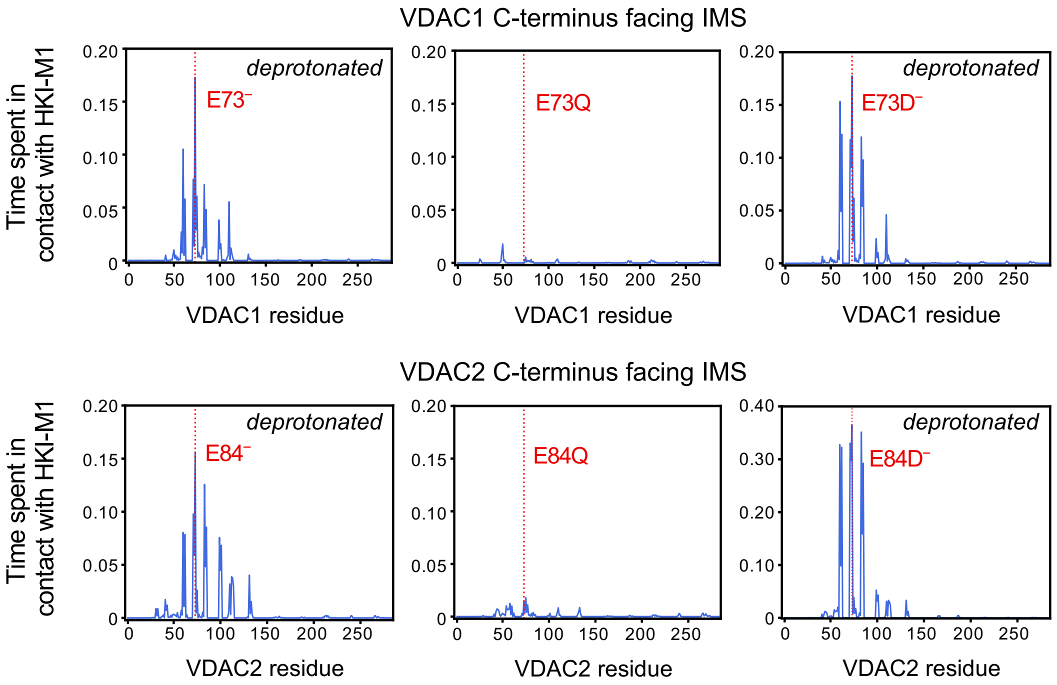

Supplementary Figure 5 | VDACs with a Glu-to-Asp substitution retain the ability to bind HKI-N. Relative duration of contacts between HKI-Met1 and specific residues of VDAC1, $VDAC1^{E73Q}$, $VDAC1^{E73D}$, VDAC2, $VDAC2^{E84Q}$ and $VDAC2^{E84D}$ with deprotonated bilayer-facing acidic residues and IMS-facing *C*-termini simulated in an OMM-mimicking bilayer. Shown are the combined data of three individual replicas with a total simulation time of 163 µs and 211 µs per condition.

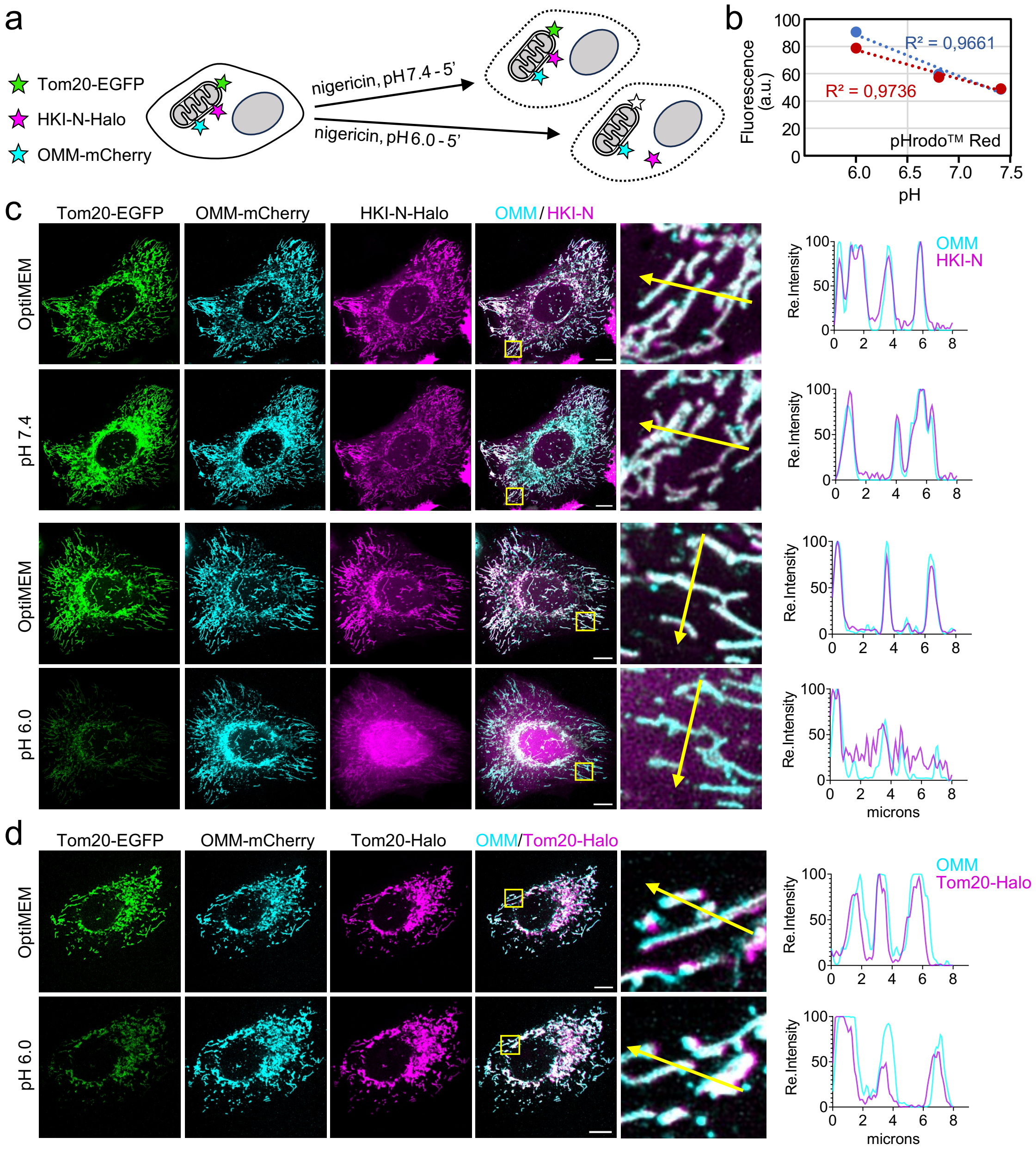

Supplementary Figure 6 | Acidification of cytosolic pH triggers dissociation of HKI-N from mitochondria. (**a**) Schematic outline of experimental strategy to determine the impact of cytosolic acidification on mitochondrial association of HKI-N. (**b**) HeLa cells were incubated with 5 μM pHrodo™ Red AM and 10 μM nigericine in buffers with descending pH (7.4, 6.8 and 6.0) for 10 min at 37°C per buffer. Relative fluorescence levels were plotted in the graph and a linear trendline was fitted to get the pH standard curve. Data shown are from two independent experiments. (**c**) Fluorescence images of live HeLa cells co-expressing EGFP-tagged Tom20 (*green*), OMM-mCherry (*cyan*) and Halo-tagged HKI-N (*magenta*) grown in Optimem (top) and then treated with nigericin in pH 7.4 buffer or pH 6.0 buffer for 5 min. Line scans showing degree of overlap between OMM and HKI-N signals along the path of the arrow shown in the zoom-in. Scale bar, 10 μm. (**d**) Fluorescence images of live HeLa cells co-expressing EGFP-tagged Tom20 (*green*), OMM-mCherry (*cyan*) and Halo-tagged Tom20 (*magenta*) grown in Optimem (top) and then treated with nigericin in pH 6.0 buffer for 5 min. Line scans showing degree of overlap between OMM and Tom20-Halo signals along the path of the arrow shown in the zoom-in. Scale bar, 10 μm.

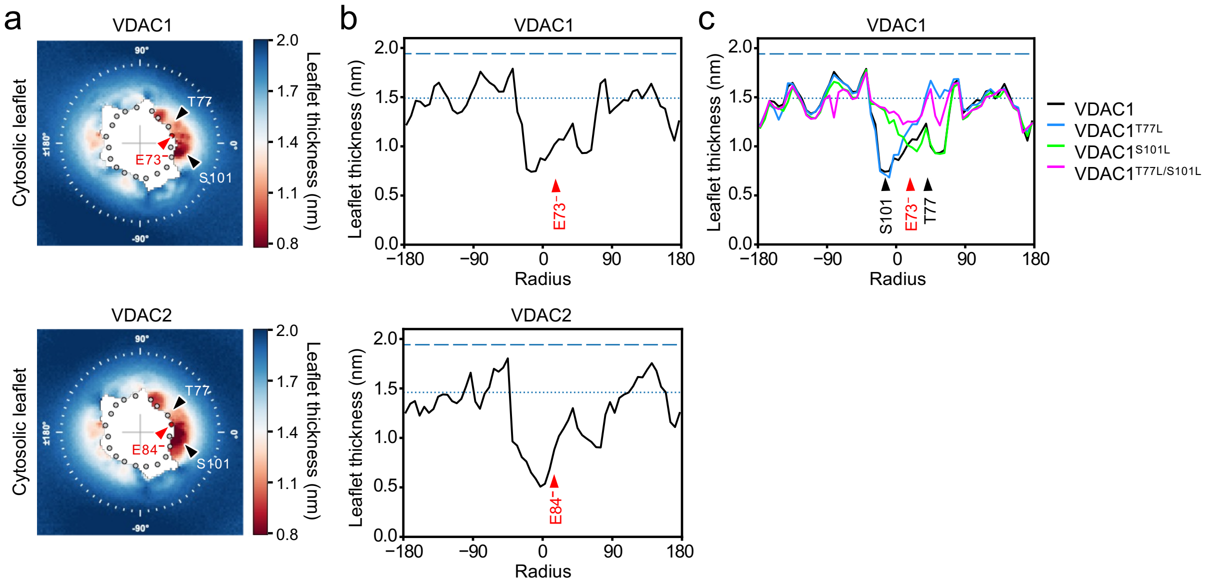

Supplementary Figure 7 | Cytosolic leaflet thickness profiles around VDAC1, VDAC1^{T77L}, VDAC1^{S101L} and VDAC1$^{T77L/S101L}$. (**a**) Diagrams reproducing the top panels of Fig. 6a of the main text, overlaid with a reference for the angles considered in (b) and (c). (**b**) Minimum measured cytosolic leaflet thickness as a function of the angle around the VDAC barrel, from the datasets in Fig. 6a. The dashed line corresponds to the average leaflet thickness in a protein-free POPC membrane (1.94±0.001 nm); the dotted line represents the average of the plotted line (1.49±0.01 nm and 1.46±0.01 nm for VDAC1 and VDAC2, respectively) excluding the defect region (-33° to 75° for VDAC1 and -39° to 81° for VDAC2). (**c**) Same measure as in (b), from the datasets in Fig. 7b. The dashed and dotted lines, and the black line, are the same as in the top panel of (b). Mutating the hydrophilic Thr77 and Ser101, first individually then simultaneously, is shown to gradually abolish thinning in the defect region close to Glu73, bringing the leaflet to thicknesses comparable to those experienced by the rest of the barrel.

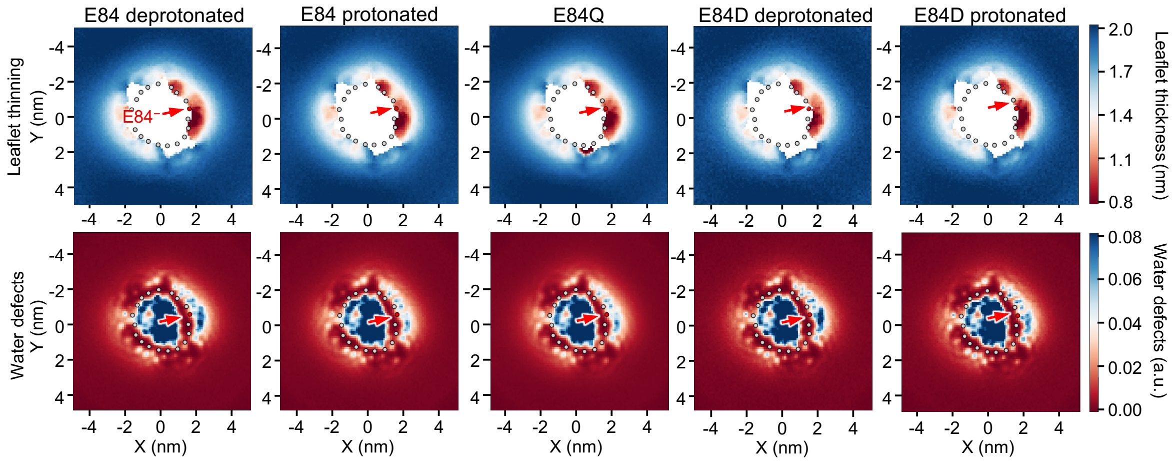

Supplementary Figure 8 | VDAC2 causes lipid packing defects and membrane leaflet thinning proximal to the bilayer-facing Glu. Cytosolic leaflet thinning and water defect graphs of VDAC2, VDAC2^{E84Q} and VDAC2^{E84D} simulated in a POPC bilayer with *C*-termini facing the IMS (bottom) leaflet. The bilayer-facing acidic residues were protonated or deprotonated, as indicated. Analysis was done as in Fig. 6a and 6b.

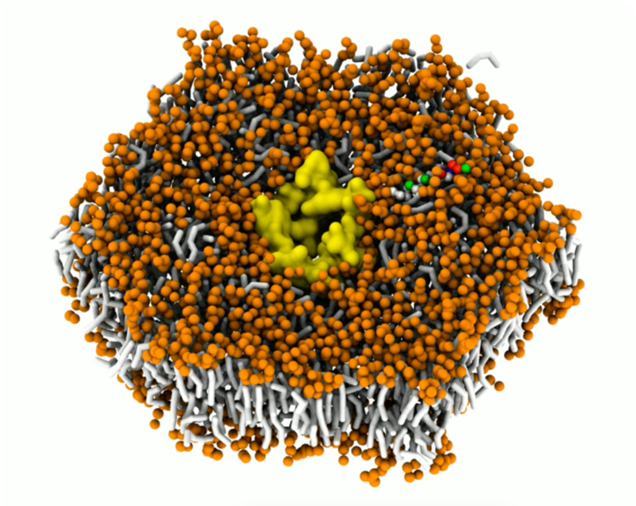

Supplementary Movie 1 | MD simulation of complex formation between HKI-N and VDAC1. Simulation of HKI-N binding to VDAC1 with a deprotonated E73 and IMS-facing *C*-terminus in an OMM-mimicking bilayer. The movie captures 849 ns of simulated time. The image is a screen shot of the first frame of the movie.

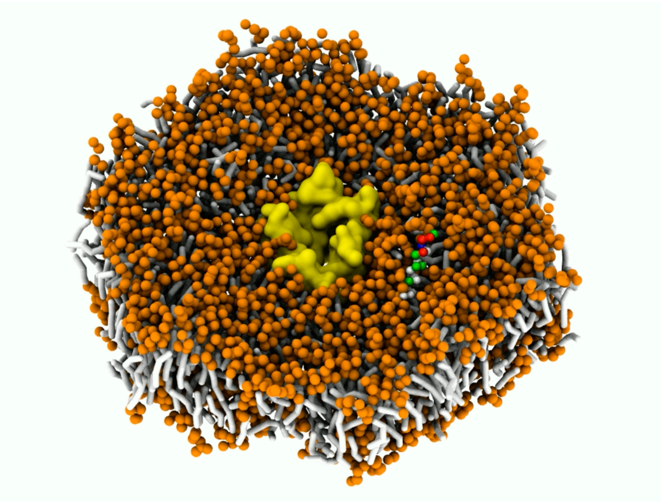

Supplementary Movie 2 | MD simulation of complex formation between HKI-N and VDAC2. Simulation of HKI-N binding to VDAC2 with a deprotonated E84 and IMS-facing *C*-terminus in an OMM-mimicking bilayer. The movie captures 1400 ns of simulated time. The image is a screen shot of the first frame of the movie.

Supplementary Table 1 | Simulated systems.

System[a]	Box vector lengths (nm)	Membrane composition	Number of replicates	Simulation time[e] (µs)
VDACs with HKI-N	16.2, 16.2, 8.9 [b]	OMM mimic[d]	3	
VDAC1 E73 deprotonated $_{COOH-IMS}$				210.9
VDAC1 E73 protonated $_{COOH-IMS}$				169.3
VDAC1 E73 deprotonated $_{COOH-cyto}$				169.4
VDAC1 E73 protonated $_{COOH-cyto}$				177.9
VDAC2 E84 deprotonated $_{COOH-IMS}$				170.3
VDAC2 E84 protonated $_{COOH-IMS}$				170.4
VDAC2 E84 deprotonated $_{COOH-cyto}$				168.9
VDAC2 E84 protonated $_{COOH-cyto}$				177.1
VDACs w/o HKI-N	11.7, 11.7, 14.9 [c]	100% POPC	3	
VDAC1 E73 deprotonated				32.4
VDAC1 E73 protonated				31.9
VDAC2 E84 deprotonated				32.6
VDAC2 E84 protonated				32.4
VDAC mutants with HKI-N	16.2, 16.2, 8.9 [b]	OMM mimic[d]	3	
VDAC1 E73Q $_{COOH-IMS}$				163.5
VDAC1 E73D deprotonated $_{COOH-IMS}$				300.4
VDAC1 E73D protonated $_{COOH-IMS}$				158.0
VDAC2 E84Q $_{COOH-IMS}$				173.4
VDAC2 E84D deprotonated $_{COOH-IMS}$				199.1
VDAC1 E73F/F71E $_{COOH-IMS}$				162.8
VDAC1 E73F/F71E $_{COOH-cyto}$				148.9
VDAC1 S101L $_{COOH-IMS}$				172.4
VDAC1 T77L $_{COOH-IMS}$				171.0
VDAC1 S101L/T77L $_{COOH-IMS}$				169.7
VDAC mutants w/o HKI-N	11.7, 11.7, 15.0 [c]	100% POPC	3	
VDAC1 E73Q				55.2
VDAC1 E73D deprotonated				49.6
VDAC1 E73D protonated				30.1
VDAC2 E84Q				59.1
VDAC2 E84D				57.2
VDAC1 E73F/F71E				26.4
VDAC1 S101L				32.6
VDAC1 T77L				27.7
VDAC1 S101L/T77L				27.4
HKI-N-only desorption tests	9.4, 9.4, 10.1 [c]	OMM mimic[d]	6	
HKI-N				23.3
HKI-N L7Q				1.3
Protein-free POPC membrane	7.0, 7.0, 7.6 [b]	100% POPC	3	31.1
**Total simulation time: 3.78 ms**				

[a] Subscripts in VDAC systems in OMM mimic membranes indicate orientational topology; the C-terminus of VDAC was oriented towards the IMS leaflet (COOH-IMS) or the cytosolic leaflet (COOH-cyto).

[b] Hexagonal prismatic unit cell.

[c] Orthorhombic unit cell.

[d] Cytosolic leaflet: POPC/POPE/SAPI/cholesterol (45/33.5/5/16.5, mol%);
IMS leaflet: POPC/POPE/SAPI/cholesterol (52.5/14/19/14.5, mol%).

[e] Simulation times are reported as the sum over the replicates for each system.

Supplementary Table 2 | Primers used in this study.

Primer name	Primer sequence (5’-3’)
pSEMS HKI-N-Halo fwd	GCTGAAGATGATGTGGAAGTGTGGTGGAATTCCATGATCG
pSEMS HKI-N-Halo rev	CCTGTGCCATGGCCCCCGTCATCCTTCAGCTCCG
pEGFP ratHKI-L7Q fwd	CGCGCAACTACAGGCCTATTACT
pEGFP ratHKI-L7Q rev	GCGATCATGCTGACGGTG
pEGFP ratHKIΔ2-14 fwd	AAGGATGACCAAGTCAAAAAGATTGACAAG
pEGFP ratHKIΔ2-14 rev	CATGCTGACGGTGGGGGA
pcDNA3.1 hVDAC1-HA fwd	AAGCTGGCTAGCACCATGGCAATGGCTGTGCCACCCACG
pcDNA3.1 hVDAC1-HA rev	GGGCCCTCTAGATCAGGCGTAATCCGGCACATCATAGGGGTATGCTTGAAATTCCAGTCC
pcDNA3.1 hVDAC2-HA fwd	AAGCTGGCTAGCACCATGGCAATGGCGACCCACGGACAG
pcDNA3.1 hVDAC2-HA rev	GGGCCCTCTAGATCAGGCGTAATCCGGCACATCATAGGGGTAAGCGTCCAACTCCAGGGC
pcDNA3.1 hVDAC1^{E73Q}-HA fwd	GGCCTGACGTTTACACAGAAATGGAATACCGAC
pcDNA3.1 hVDAC1^{E73Q}-HA rev	GTCGGTATTCCATTTCTGTGTAAACGTCAGGCC
pcDNA3.1 hVDAC2^{E84Q}-HA fwd	GGTCTGACTTTCACACAAAAGTGGAACACTGATAAC
pcDNA3.1 hVDAC2^{E84Q}-HA rev	GTTATCAGTGTTCCACTTTTGTGTGAAAGTCAGACC
pcDNA3.1 hVDAC1^{E73D}-HA fwd	GACAAATGGAATACCGACAATACACTAG
pcDNA3.1 hVDAC1^{E73D}-HA rev	TGTAAACGTCAGGCCGTACTCAG
pcDNA3.1 hVDAC2^{E84D}-HA fwd	GACAAGTGGAACACTGATAACACTCTG
pcDNA3.1 hVDAC2^{E84D}-HA rev	TGTGAAAGTCAGACCATACTCAC
pcDNA3.1 hVDAC1$^{E73F/F71E}$-HA fwd	GAGACATTTAAATGGAATACCGACAATACACTAG
pcDNA3.1 hVDAC1$^{E73F/F71E}$-HA rev	CGTCAGGCCGTACTCAGTCC
pcDNA3.1 hVDAC1^{T77L}-HA fwd	CTGGACAATACACTAGGCACCGAG
pcDNA3.1 hVDAC1^{T77L}-HA rev	ATTCCATTTCTCTGTAAACGTCAG
pcDNA3.1 hVDAC1^{S101L}-HA fwd	CTGTCCTTCTCACCTAACACTGGG
pcDNA3.1 hVDAC1^{S101L}-HA rev	ATCGAAGGTCAGCTTCAGTCC
pcDNA3.1 (+) VDAC1$^{T77L/S101L}$-HA fwd	CTGGACAATACACTAGGCACCGAG
pcDNA3.1 (+) VDAC1$^{T77L/S101L}$-HA rev	ATTCCATTTCTCTGTAAACGTCAG

Supplementary Table 3 | Chromatic shifts in reference to GFP channel

Shift	mCherry	JF646
Dx (µm)	-0.06	-0.131
Dy (µm)	-0.006	0.017
Dz (µm)	-0.314	-0.648
Rot (°)	0.034	-0.038
Scale	0.999	1.001

Image J Macro

<u>Pearson's analysis</u>

```
dir1 = getDirectory("Choose Source Directory");
list = getFileList(dir1);
counter = 0;
var channel, slice, frame;

for (i=0; i<list.length; i++) {
  filename = dir1 + list[i];
  //check if the current file is in fact a subfolder
  if (File.isDirectory(filename)) {
  }
  else {
    if (endsWith(filename, ".dv")) {
      run("Bio-Formats Importer", "open=["+filename+"] autoscale color_mode=Default 
rois_import=[ROI manager] view=Hyperstack stack_order=XYCZT");
      selectImage(list[i]);
      run("Maximize");
      Stack.setPosition(3, 8, 0);
      setTool("polygon");
      waitForUser("Z and ROI", "Select a z-plane and draw ROI - polygon tool is 
already selected! Then click 'Ok'");
      setBatchMode(true);
      Stack.getPosition(channel, slice, frame);
      run("Crop");
      setBackgroundColor(0, 0, 0);
      Stack.setChannel(3);
      run("Duplicate...", "  title=channel2 channels=3 slices="+slice);
      run("Clear Outside");
      selectImage(list[i]);
      Stack.setChannel(1);
      run("Duplicate...", "  title=channel3 channels=1 slices="+slice);
      run("Clear Outside");
      close(list[i]);
      run("JACoP ", "imga=channel2 imgb=channel3 pearson costesthr");
      dataStr = split( getInfo("Log"), "\n" );
      res = substring( dataStr[5], indexOf( dataStr[5], "=" ) + 1, lengthOf( 
dataStr[5] ) );
      costes = substring( dataStr[9], indexOf( dataStr[9], "=" ) + 1, indexOf( 
dataStr[9], "(" ) - 1);
      print("\\Clear");
      setResult("Filename", counter, list[i]);
      setResult("Pearson", counter, res);
      setResult("Costes", counter, costes);
      close("channel2");
      close("channel3");
      close('Costes*');
      counter = counter+1;
      setBatchMode(false);
    }
  }
}
selectWindow("Log");
run("Close");
```

Supplementary reference

1. Dadsena, S. *et al.* Ceramides bind VDAC2 to trigger mitochondrial apoptosis. *Nat. Commun.* **10**, (2019).

Unprocessed images of immunoblots

Supplementary Figure 2c - uncropped

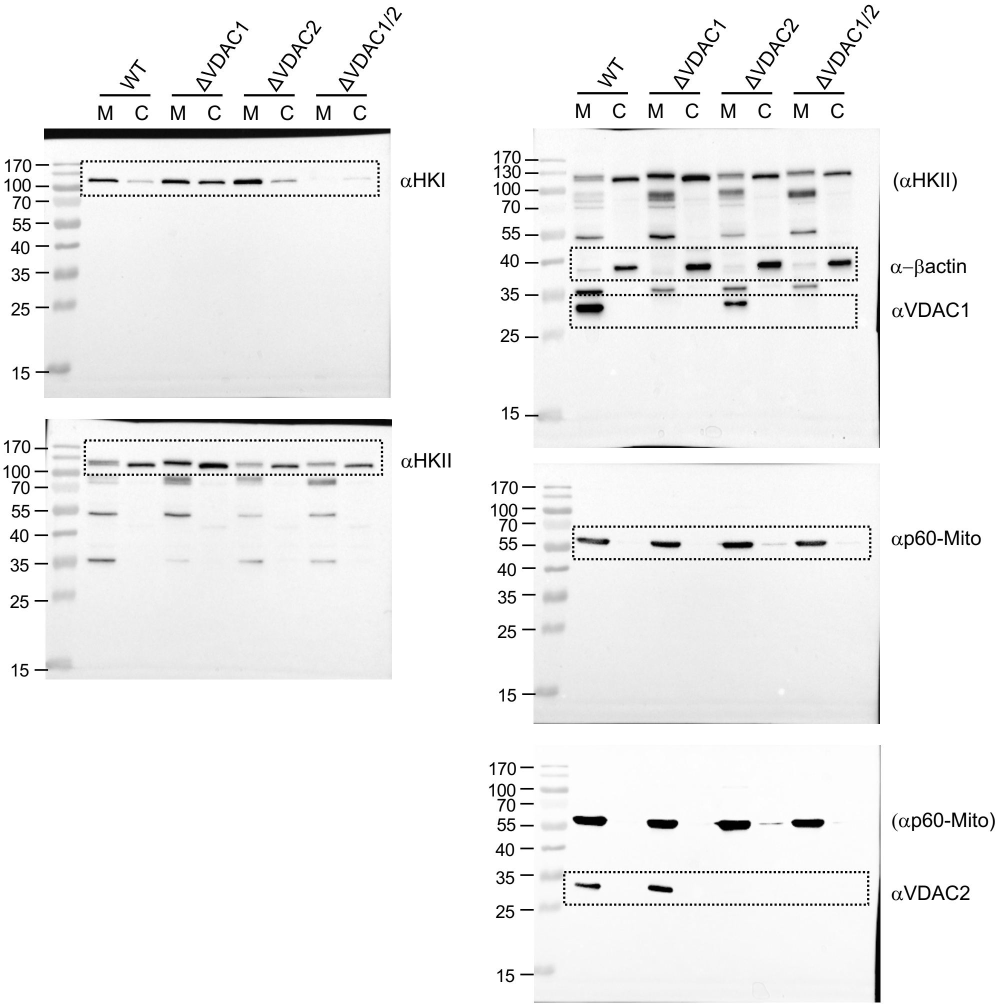

Supplementary Figure 2d - uncropped

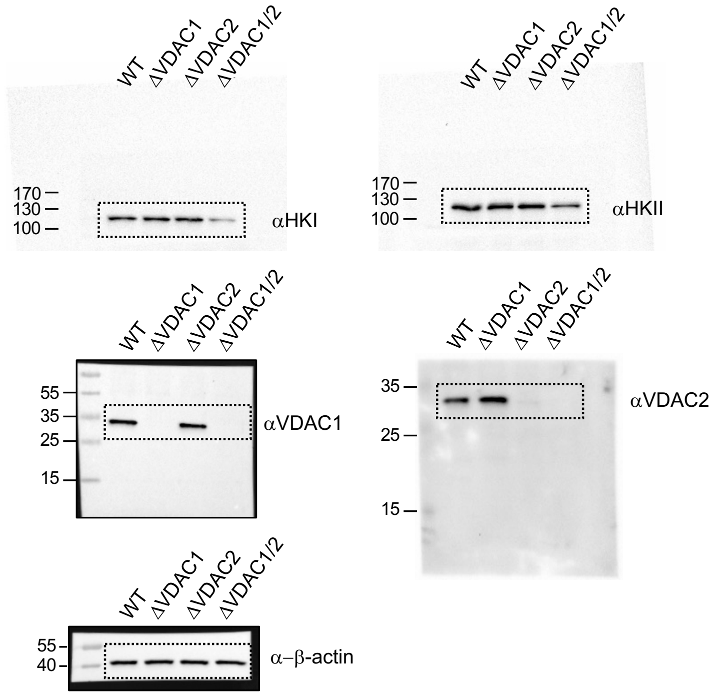

Supplementary Figure 2e - uncropped

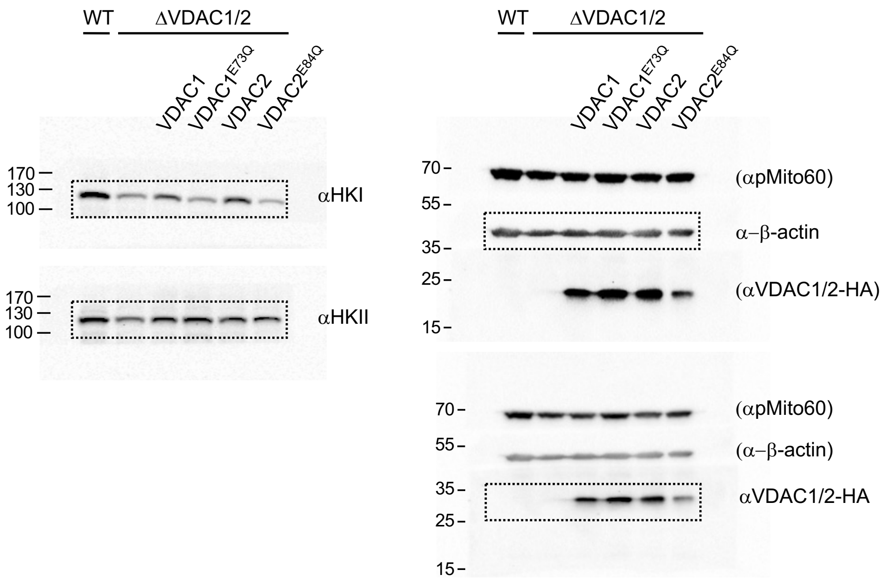

Supplement: Supplementary file 1 — Supplementary Information [file 42003_2025_7551_MOESM1_ESM.pdf]
