# Supplementary material for: Hexokinase-I directly binds to a charged membrane-buried glutamate of mitochondrial VDAC1 and VDAC2
Source: Commun Biol. 2025 Feb 10;8:212. doi: 10.1038/s42003-025-07551-9 (PMC11811193; doi:10.1038/s42003-025-07551-9)
Supplement: Supplementary file 2 — Description of Additional Supplementary Files [file 42003_2025_7551_MOESM2_ESM.pdf]

# Description of Additional Supplementary Files

File name: Supplementary Movie 1.

Description: MD simulation of complex formation between HKI-N and VDAC1.

File name: Supplementary Movie 2.

Description: MD simulation of complex formation between HKI-N and VDAC2.

File name: Supplementary Data.

Description: The source data behind all graphs in the paper.
