# Supplementary material for: Hexokinase-I directly binds to a charged membrane-buried glutamate of mitochondrial VDAC1 and VDAC2
Source: Commun Biol. 2025 Feb 10;8:212. doi: 10.1038/s42003-025-07551-9 (PMC11811193; doi:10.1038/s42003-025-07551-9)
Supplement: Supplementary file 6 — Reporting Summary [file 42003_2025_7551_MOESM6_ESM.pdf]

nature portfolio

Corresponding author(s): Joost C. M. Holthuis

Last updated by author(s): Jan 2, 2025

Reporting Summary

Nature Portfolio wishes to improve the reproducibility of the work that we publish. This form provides structure for consistency and transparency in reporting. For further information on Nature Portfolio policies, see our Editorial Policies and the Editorial Policy Checklist.

Statistics

For all statistical analyses, confirm that the following items are present in the figure legend, table legend, main text, or Methods section.

n/a	Confirmed	
☐	☒	The exact sample size (*n*) for each experimental group/condition, given as a discrete number and unit of measurement
☐	☒	A statement on whether measurements were taken from distinct samples or whether the same sample was measured repeatedly
☐	☒	The statistical test(s) used AND whether they are one- or two-sided<br>*Only common tests should be described solely by name; describe more complex techniques in the Methods section.*
☒	☐	A description of all covariates tested
☒	☐	A description of any assumptions or corrections, such as tests of normality and adjustment for multiple comparisons
☐	☒	A full description of the statistical parameters including central tendency (e.g. means) or other basic estimates (e.g. regression coefficient) AND variation (e.g. standard deviation) or associated estimates of uncertainty (e.g. confidence intervals)
☐	☒	For null hypothesis testing, the test statistic (e.g. *F*, *t*, *r*) with confidence intervals, effect sizes, degrees of freedom and *P* value noted<br>*Give P values as exact values whenever suitable.*
☒	☐	For Bayesian analysis, information on the choice of priors and Markov chain Monte Carlo settings
☒	☐	For hierarchical and complex designs, identification of the appropriate level for tests and full reporting of outcomes
☒	☐	Estimates of effect sizes (e.g. Cohen's *d*, Pearson's *r*), indicating how they were calculated

Our web collection on statistics for biologists contains articles on many of the points above.

Software and code

Policy information about availability of computer code

Data collection

The Martini 3 force field was used for all CG-MD simulations and the secondary structure of all proteins was restricted using an elastic-network approach, placing harmonic bonds of 500 kJ mol-1 nm-2 between backbone particles that lie within 0.9 nm after coarse-graining of the reference structures. All simulations were run with GROMACS versions 2021.

Titratable Martini simulations of VDAC channels were performed using the stochastic dynamics integrator with a timestep of 10 fs. The PME algorithm was used to calculate electrostatic interactions, with a cutoff value of 1.1 nm. For analysis, only the last 10 ns of each simulation were considered. The scripts to perform the titrations and analysis can be found at https://github.com/fgrunewald/titratable_martini_tools and http://cgmartini.nl.

Live cells were imaged using a Zeiss Cell Observer microscope equipped with a CSU-X1 spinning disk unit (Yokogawa) at 37°C. Images were acquired at magnification of 75.6 x using an Alpha Plan-Apochromat 63x oil immersion objective (NA 1.46) and immersion oil for 37°C (Immersol 518 F, 1.518, Zeiss).

Immunostained cells were imaged using a DeltaVision Elite microscope (GE Healthcare) using a PLAPON 60x oil immersion objective (NA 1.42) and Immoil FC30CC immersion oil (Olympus Life Science, n= 1.518, 23°C).

Data analysis

Analysis algorithms for CG-MD simulations were programmed in Python, using MDAnalysis (https://www.mdanalysis.org) and NumPy packages. The MDreader package (https://github.com/mnmelo/MDreader) was used to allow analysis parallelization, which was essential in tackling the multi-gigabyte trajectory analysis in a short time. Visual molecular dynamics (VMD) software was used to create images and videos.

Analysis of microscopy images were performed on the original, unmodified data using Fiji Image J2 software (version 2.3.0/1.54i) and Image J Macros provided in Supplementary Information. Deltavision images were deconvoluted using SoftWoRx 5.5 software and further processed using Fiji software.

For manuscripts utilizing custom algorithms or software that are central to the research but not yet described in published literature, software must be made available to editors and reviewers. We strongly encourage code deposition in a community repository (e.g. GitHub). See the Nature Portfolio guidelines for submitting code & software for further information.

Data

Policy information about availability of data

All manuscripts must include a data availability statement. This statement should provide the following information, where applicable:

- Accession codes, unique identifiers, or web links for publicly available datasets
- A description of any restrictions on data availability
- For clinical datasets or third party data, please ensure that the statement adheres to our policy

All data generated or analyzed in this study are included in the manuscript and supporting files. Source data with sample sizes, number of technical and/or biological replicates, means, standard deviations, and calculated p values (where applicable) are provided in the Supplementary Data file. Uncropped scans of immunoblots are provided in Supplementary Information.

Research involving human participants, their data, or biological material

Policy information about studies with human participants or human data. See also policy information about sex, gender (identity/presentation), and sexual orientation and race, ethnicity and racism.

Reporting on sex and gender	N/A
Reporting on race, ethnicity, or other socially relevant groupings	N/A
Population characteristics	N/A
Recruitment	N/A
Ethics oversight	N/A

Note that full information on the approval of the study protocol must also be provided in the manuscript.

Field-specific reporting

Please select the one below that is the best fit for your research. If you are not sure, read the appropriate sections before making your selection.

☒ Life sciences ☐ Behavioural & social sciences ☐ Ecological, evolutionary & environmental sciences

For a reference copy of the document with all sections, see nature.com/documents/nr-reporting-summary-flat.pdf

Life sciences study design

All studies must disclose on these points even when the disclosure is negative.

Sample size	Sample size for each experiment is indicated in the figure. No statistical method was used for sample predetermination. Sample sizes were determined based on the author's experience of what is necessary to generating a convinving and compelling result.
Data exclusions	No data were excluded from the analyses of the experiments.
Replication	All experimental findings were reliably reproduced at least once, using independent experimental samples.
Randomization	Plates of wells with cultured cells were randomly assigned to experimental groups. Microscope image acquisition was performed randomly.
Blinding	No blinding was done in this study. Virtually all the data are quantitative. Most measurements are made using a machine and not easily subjected to operator bias.

Reporting for specific materials, systems and methods

We require information from authors about some types of materials, experimental systems and methods used in many studies. Here, indicate whether each material, system or method listed is relevant to your study. If you are not sure if a list item applies to your research, read the appropriate section before selecting a response.

Materials & experimental systems

n/a	Involved in the study
☐	☒ Antibodies
☐	☒ Eukaryotic cell lines
☒	☐ Palaeontology and archaeology
☒	☐ Animals and other organisms
☒	☐ Clinical data
☒	☐ Dual use research of concern
☒	☐ Plants

Methods

n/a	Involved in the study
☒	☐ ChIP-seq
☒	☐ Flow cytometry
☒	☐ MRI-based neuroimaging

Antibodies

Antibodies used

Mouse monoclonal anti-Tom20 (Millipore, MABT166, clone 2F8.1, IF 1:200), mouse monoclonal anti-mitochondrial surface protein p60 (Millipore, MAB1273, IB 1:1,000), rabbit polyclonal anti-HA (Invitrogen, 71-5500; clone SG77, IF 1:100), rabbit polyclonal anti-HKI (Cell Signaling, 2024-s, IB 1:1000), rabbit polyclonal anti-HKII (Cell Signaling, 2867-s, IB 1:1,000), rabbit monoclonal anti-VDAC1 (Cell Signaling, 4661-s, IB 1:1,000), goat polyclonal anti-VDAC2 (Abcam, ab37985, IB 1:4,000), mouse monoclonal anti-β-actin (Sigma, A1978, IB 1:50,000), CyTM-dye-conjugated donkey anti-mouse (Jackson ImmunoResearch Europe Ltd, 715-225-150, 715-162-150 and 715-175-150; IF 1:200 each), donkey anti-rabbit (Jackson ImmunoResearch Europe Ltd, 711-225-152, 711-175-150 and 711-165-150; IF 1:200 each), HRP-conjugated goat anti-mouse IgG (Thermo Fisher Scientific; 31430; IB 1:5,000), HRP-conjugated goat anti-rabbit IgG (Thermo Fisher Scientific; 31460; IB 1:5,000), HRP-conjugated donkey anti-goat IgG (Thermo Fisher Scientific, PA1-28664; IB 1:5,000).

Validation

Specificity of anti-VDAC and anti-HKI antibodies was verified by immunoblot analysis of total lysates from VDAC-KO and HKI-KO cells as documented in the MS. All other commercial antibodies were validated by the suppliers as follows:

Mouse monoclonal anti-Tom20 (Millipore, MABT166, clone 2F8.1)
https://www.merckmillipore.com/DE/en/product/Anti-Tom20-Tomm20-Antibody-clone-2F8.1,MM_NF-MABT166

Mouse monoclonal anti-mitochondrial surface protein p60 (Millipore, MAB1273)
https://www.merckmillipore.com/DE/en/product/Anti-Mitochondria-Antibody-surface-of-intact-mitochondria-clone-113-1,MM_NF-MAB1273

Rabbit polyclonal anti-HA (Invitrogen, 71-5500; clone SG77)
https://www.thermofisher.com/antibody/product/HA-Tag-Antibody-clone-SG77-Polyclonal/71-5500

Rabbit polyclonal anti-HKII (Cell Signaling, 2867-s)
https://www.cellsignal.com/products/primary-antibodies/hexokinase-ii-c64g5-rabbit-mab/2867

Mouse monoclonal anti-β-actin (Sigma, A1978)
https://www.sigmaaldrich.com/NL/en/product/sigma/a1978

HRP-conjugated goat anti-mouse IgG (Thermo Fisher Scientific; 31430)
https://www.thermofisher.com/antibody/product/Goat-anti-Mouse-IgG-H-L-Secondary-Antibody-Polyclonal/31430

HRP-conjugated goat anti-rabbit IgG (Thermo Fisher Scientific; 31460)
https://www.thermofisher.com/antibody/product/Goat-anti-Rabbit-IgG-H-L-Secondary-Antibody-Polyclonal/31460

Cyanine Cy™2-conjugated donkey anti-mouse IgG (Jackson ImmunoResearch Laboratories, 715-225-150)
https://www.jacksonimmuno.com/catalog/products/715-225-150

Cyanine Cy™2-conjugated donkey anti-rabbit IgG (Jackson ImmunoResearch Laboratories, 711-225-152)
https://www.jacksonimmuno.com/catalog/products/711-225-152

Cyanine Cy™3-conjugated donkey anti-mouse IgG (Jackson ImmunoResearch Laboratories, 715-162-150)
https://www.jacksonimmuno.com/catalog/products/715-162-150

Cyanine Cy™3-conjugated donkey anti-rabbit IgG (Jackson ImmunoResearch Laboratories, 715-165-152)
https://www.jacksonimmuno.com/catalog/products/715-165-152

Cyanine Cy™5-conjugated donkey anti-mouse IgG (Jackson ImmunoResearch Laboratories, 715-175-150)
https://www.jacksonimmuno.com/catalog/products/715-175-150

Cyanine Cy™5-conjugated donkey anti-rabbit IgG (Jackson ImmunoResearch Laboratories, 711-165-150)
https://www.jacksonimmuno.com/catalog/products/711-165-150

Eukaryotic cell lines

Policy information about cell lines and Sex and Gender in Research

Cell line source(s)	Human cervical carcinoma HeLa cells (ATCC CCL-2); human colon carcinoma HCT116 cells (ATCC CCL-247); human embryonic kidney HEK293T cells (ATCC CRL-3216).
Authentication	Cell lines were routinely examined for their morphology and analyzed for their characteristic protein expression profiles.
Mycoplasma contamination	All cell lines were free of mycoplasma contaminations as determined routinely by DAPI staining or PCR assay.
Commonly misidentified lines (See ICLAC register)	No commonly misidentified cell lines were used.

Plants

Seed stocks	N/A
Novel plant genotypes	N/A
Authentication	N/A
